# Forty-first Annual Meeting of the Mississippi Valley Association of Dental Surgeons

**Published:** 1885-05

**Authors:** E. G. Betty


### THE DENTAL REGISTER.

VOL. XXXIX.] MAY, 1885. [No. 5.

### PAPERS, DISCUSSIONS AND PROCEEDINGS

### Of the Forty-First Annual Meeting of the Mississippi Valley Association of Dental Surgeons.

Held at Cincinnati, O., March 4th, 5th, and 6th, 1885.

REPORTED BY E. G. BETTY, D.D.S.

*Second Day—Afternoon Session.*

The meeting was called to order promptly on time, and one hour was consumed in the exhibition, by Dr. W. S. Howe, of a new battery for running the electric mouth-lamp, mallet, etc., and by Dr. McKellops with the Bonwill automatic mallet for the engine.

Dr. A. O. Rawls then read the following paper on Pyorrhoea Alveolaris:

A question is not to be questioned, when all agree. But many dissenters may quickly evolve the truth.

A brief review of the opinion of others, relating more especially to doubtful points in connection with this disease, will probably assist our researches, and better enable us to sift the facts from the tangled mass of diverse expression.

When notice of this disease as one distinct from other inflammatory lessions of the oral cavity was first brought prominently before the dental public, scores, aye, hundreds of the profession had observed as its cause only the deposition of salivary calculus, and naturally enough the great burden of their evidence favored the theory that its origin was strictly local. But from the time Dr. Rehwinkel, of Chillicothe, and myself, saw fit to oppose this view of its causation at a meeting of the American Dental Association in Chicago, the opinions have very materially changed, and to-day finds the number of advocates of each theory (*viz.*, the local or systemic) pretty evenly balanced.

The local cause theorists are at variance in their opinions as to whether the cause be of this or that nature, some claiming salivary calculus as its origin, a limited number sanguinary calculus, and still others whose observations so far lead them to the belief that the affection is resultant from "a peculiar organism or fungus growth which always fills the sockets." To Dr. Riggs, of Hartford, I think is justly due much for the impetus given the investigation by the profession in this matter, and upon him I look as the pioneer of the local origin theory. If I am not misinformed, Dr. Riggs claims that salivary calculus is the immediate causation with no remoteness about it, and his treatment, so far as I have been able to discover, is certainly consistent with such views, besides being far superior in my estimation to any other local treatment resorted to by either set of theorists.

Dr. Ingersoll, of Iowa, has given evidence of the fact that he is a searcher for the truth at the bottom, announcing the theory of sanguinary calculus as a cause, stating that often when no salivary deposits were present, the sanguinary calculus would be found far up the roots of the teeth, which latter, I think, correct, but as to the name would prefer that in more general use, *viz.*, serumal calculus.

Our esteemed friend, Dr. Black, of Illinois, next comes to the front and presents the latest local origin theory in support of which, and confirmitory of his microscopic investigations, he has the results of similar research at the hands of Dr. Witzell, of Germany, whose article bearing upon the subject was somewhat previous to Dr. Black's.

This is one of the numerous germ theories and is founded upon the fact of the presence in these cases, of little hot-beds of peculiar organisms or fungi, which, according to supporters of the theory, cause a melting away of the pericemental membrane; by what means is not definitely understood but presumably by the "digestion fluids of the fungus growth, causing a re-moleculization of tissues exposed to their action." And now in behalf of the subject from another standpoint and as I understand it.

The characteristic symptoms and signs of this much dreaded malady are somewhat varying in their nature. The progress of the disease also differs in accordance with formation of the parts, habits of the patient, environment, nature of cause and whether the latter be hereditary or acquired.

The incipient symptoms are to a casual observer, seldom noticed as such, simply for the reason that they are in their expressions somewhat analagous to those occurring in pericementitis or submucous irritation from ordinary causes.

Dr. Black said the disease is first rendered apparent by a red line at the border of the gum and asserts his belief in this as a constant first symptom. Now while this may be true as it refers to occasional first apparent signs, it is according to my observation no part of the truth as it refers to first symptoms.

First symptoms are not seen, they are felt.

About the earliest subjective symptom is either a sense of fullness or a feeling of impactedness in the vicinity of such teeth as may be involved.

Why there should be a difference in incipient symptoms in different cases or varying localities in the same mouth may be, and probably is, on account of difference in the shape of the teeth, the direction of contact and point of initial lesion. The above symptoms are usually followed by more or less soreness and springy feeling under delicate percussion, the soreness gradually lessening as the disease progresses until the lesions become so extensive as to admit the action of deposited irritants, when it again from time to time returns and abates alternately as these parts are relieved by cleansing and depletion or congested by close contact with irritating deposits.

Following these primary symptoms come the more pronounced (though of difficult detection) objective symptoms and signs, the first of which is not "a red line at the border of the gum," but a slight prominence or thickening at that point, with no perceptible change in color from that normal to the part. In not a few cases the foregoing manifestations are successively present and yet no deposits of a calcareous character to be seen or felt and occasionally not even a disruption of the tissues at the neck of the tooth or teeth thus implicated. At this phase of the disease, our friend "red line" may follow, though generally not until there has been present a slightly bluish or purplish tint which is indicative of increased size of the capillaries, congestion, and carbonization of the blood at the point referred to; this color passes away with disruption of the tissues implicated and is gradually replaced with a more angry looking red line, though not always, especially is it not so, when the lesion exists between the teeth.

Following these symptoms and signs, the dissolution of continuity continues between gum and tooth; the objective symptoms become more marked with the influx, a deposit of additional irritants and in turn the periosteum at the margin of the process or over the septa between the teeth is laid bare and subjected to the exciting causes of dissolution, which had induced its exposure. The destruction of peri-dentium advances toward the apex of the root, the periosteum melts away, there is a breach in the round of nutrient circulation that had once supplied the alveolar border, and the work of disintegration proceeds in the wake of decreasing nutrition.

Shortly after the process begins to soften, the external indications will vary in different cases. There may exist a dark turgidity of the gum, or the latter may be absent entirely, and in its stead a more or less bluish red line showing the depth and direction of the dissolution; this latter only when the attack is on the posterior or anterior surface of the root; again, neither of the foregoing signs will prevail and nothing but a slightly raised, thickened, and loosened condition of the gum at its margin would indicate anything wrong. When the latter signs obtain, especially if but little pus can be pressed out, it is usually indicative of greater integrity of tissue, and if occuring in cases of heredity records a decline in the system of the remote cause or predisposition.

The points which are usually first attacked are such as might naturally be expected by any close observer, where there exists the greatest amount of gum tissue with the least of external contact or exercise, and about the openings of the ducts of the several salivary glands. This, 'tis true, gives color more or less to the local cause theory, of which, more anon. Other points of attack are such as may result from peculiarly shaped or irregularly placed teeth in different parts of either or both maxillæ, such as molars or biscuspids that have pressed forward or been pushed backward out of position, broad square-crowned molars and bicuspids with very narrow necks, showing depressions either longitudinal or transverse, lateral incisors standing in from the line of the arch which the position of the cuspids would indicate broad cutting-edges and narrow necks of the incisors. The twisting or mal-position of any one or number of the teeth which would favor the existence of an area of soft tissue comparatively unexercised, either by the act of mastication, the tongue, muscles of contiguous parts or efforts at cleansing.

As previously stated, the progress of this disease varies according to formation of parts, habits of the patient, environment, nature of cause, and whether the latter be inherited or acquired. We have just observed how much the formation of parts affects their suscepibility to initial lesions. So in like manner does it influence more or less the direction of destruction as well as the rapidity with which it takes place.

In cases where the alveolar ridge is thin from posterior to anterior surface the progress of the disease is likely to be continuous in one direction, *viz:* from the point where the process is first denuded of its membrane, directly toward the apex of the root. Although the attack in such cases occasionally takes place on the anterior or posterior margins of the border, it is of more frequent occurrence upon the septum between the teeth, often giving evidence of little or no breaking down of the septum but destroying the pericementum in a straight line toward the end of the root. Should the septum also be very thin between the teeth involved, and especially if this condition were present nearly the length of the root, it would be comparatively tenacious of life and slow to give way, this arising probably from the fact of its greater density as compared with thicker septa ; and if the disease attacked but one side, to its close association with anastamosing nutrient currents of the adjoining tooth. Upon the other hand, should the process be heavy, square, thick, and of considerable depth, a different action may be looked for in this, that the progress of disorganization will be of slower approach in a direct line toward the end of the root, and much more rapid upon the entire superficial area of the intervening septum, but while the latter is succumbing to the disease the anterior and posterior plates of the process will remain standing for a much longer period of time.

The gums in such instances, especially if the patient be ordinarily cleanly about his teeth, will, particularly in the vicinity of the molars, bicuspids, and superior incisors, drop in over the border of the posterior and anterior alveolar plates, thus presenting a flattened or somewhat squared aspect with open depressions between the teeth. Again, in case the alveolar ridge is of extraordinary width, and at the same time of but little depth, the destruction of bone proceeds in about the same manner as in the foregoing case, but if from the septum, compromises much more readily the anterior and posterior attachments of the teeth, *vice versa* if proceeding from the latter points. Instead, however, of of the gum becoming depressed between the teeth as in the foregoing instance, which similar thickness of the process would lead us to suspect, it seems to fill up the space caused by disintegration of the underlying bone, and still retains about the same relative position to the necks of the teeth as in health, and yet prevents a more generally inflammed appearance. You will observe so far as the process is concerned that in all the above cases the principal action of the disease is upon the more spongy, consequently less resisting structure between the anterior and posterior plates of the process.

When beginning on the process between the teeth the wasting away continues in various ways mentioned until the outer plates are reached, which latter have thus far under the nutrient supply of tissues external and still intact, held their own, but must now with the advance of the enemy upon the territory of their life current, go the way of the less resistive tissue they once enclosed and of which they were a part.

That daily conduct or habit of the patient has much to do with influencing the progress of this disease is certainly patent to all who have any knowledge whatever of its characteristics, and although effects thus arising may be scarcely noticeable in their bearings upon the predisposition, they evidently must according to all phisological law prevail. The effects of habit, however, to increase or lessen the amount and impress of local irritants are readily seen. Irritants of any nature tend to increase the demolition of parts, especially those denied the protection of their natural covering, hence, scrupulous care in freeing and as much as possible on the part of the patient, keeping free such parts, of substances so acting, will retard in a degree the destruction that would otherwise ensue. Again, if patients are accustomed to wholesome outdoor exercise such as more frequently induces the desire for good substantial food, consequently insuring abundant and vigorous exercise of the jaws and teeth, the disease will progress much slower than if on the other hand they subject themselves to close confinement indoors and live almost entirely upon soft pultaceous diet.

As an evidence that this matter of exercise on the parts involved is an important factor in the management of the malady, witness the difference between cases where chewing tobacco is constantly used, and those where not in use or the difference in the progress of the disease about the teeth in the same mouth, it always being in favor of the side most in use either from tobacco or food.

Possibly, the narcotic properties of tobacco may to some degree lessen the intensity of the trouble, but I apprehend the differences apparent are due more to the mechanical effects in exercising and cleansing the teeth of food, rather than to any thereapeutic action emanating from the tobacco *per se*.

To express so far the opinion that environment has much to be responsible for in connection with this condition may seem like going beyond the legitimate pale of inquiry, nevertheless if my observations go for anything, it has in a degree laid the foundation for the disease in question.

It has caused conditions which have in some manner brought about the use of mercury to ptyalism by thousands of our people. In the early settlement of each State as the "Star of

Empire westward took its way" so indeed did the blight of mercury hold its sway, and the children of those people whose principal remedial agents for almost every hidden or serious ailment were antimony, mercury, or blood-letting, are to-day, under the absolute laws of heredity, more or less the victims of parental environment.

Again, it may appear strange to you that chloride of sodium, an article of seasoning, will under certain, to me unknown conditions of the system, produce similar if not identical symptoms in the oral cavity, as may be found resultant from the use of mercury.

You ask what has this to do with environment? Well; go and make inquiry of the people who dwell midst the swamps and marshy lowlands of malarious districts, question and notice those who are the subjects of malarial posion and learn for yourself their cravings for, and extraordinary use of salt, and then observe the results upon the oral tissues.

Should you conclude that other causes, such as the presence of miasmatic germs afloat in the atmosphere have caused the lesions you will find in many mouths, then I will ask you to investigate the cases of the common sailor of the high seas, whose diet consists largely of salt meats, and you may be satisfied that salt is a factor in the production of this disease.

If still in doubt extend your researches to the common Irish laborer on our public works who has been in this country for several years and find there the effects of a long use of briny meats.

Not only does it seem true that malarious districts are among the favorite places to note the inception of this scourge but the very atmosphere of its surroundings is abundantly fruitful of germ life, and the gaping wounds or lesions of any character whether Pyorrhoea Alveolaris or otherwise will not be the less gaping for the influx of these much abused puncts of vivication, to say nothing of their offspring in such a habitat as would be their lot in not a few mouths.

Generally speaking, the acquired conditions resulting either from the use of mercury or salt are slow to manifest themselves. The effects of the former may indeed quickly produce ptyalism but the objective symptoms of the latter soon pass away, especially if the patient be of vigorous constitution and no signs characteristic of the disease be present for several years, but as time creeps on, gradually the signs become apparent, and by the time a number of years varying according to unknown influences from ten to thirty has passed, and so continues until the teeth loosen and are beyond redemption.

The majority of cases of which I now speak, you will find among people who have passed the meridian of life and on up to old age, seldom if ever in youth or early manhood. And how often do you hear the children of these afflicted ones say "why father is sixty-five years of age and has not a decayed tooth in his mouth," and then reflectively adding "but his teeth are awfully loose," and indeed the children are right for decay seldom, if ever, takes place in the teeth of persons so affected, and if perchance it existed before those condtions were acquired it seems to have stopped right there and remained in *statu quo* through life. But mark you the difference, under the laws of heredity these conditions of the parent are transmitted to the offspring, and the signs and symptoms are apparent in childhood and youth instead of old age. If however, a child is born to these parents before the conditions are acquired, no indications of the disease will be present, but on the other hand, and as sure as laws of heredity obtain to mark the features or color of the eye and hair, so certainly will the youngest children get the effects of them in the receipt of the selfsame conditions more than do the older ones. When inherited and in the first transmission, the disease is much more difficult of control, the tissues of the parts of less integrity melting rapidly away, and the attack usually more precipitous, uniform, and general throughout the mouth, though the gums of young patients retain their position comparatively well about the necks of the teeth long after the cancelous bony structure of the process has wasted away.

The varying progress of the manifestations arising from the two different causes, I believe to be noticable. True, however, I have not met with and studied a sufficient number of cases attributable to each cause to be quite so well founded in my belief upon this point as to admit no doubt. But such experience as I have had leads me to the conclusion that the disease growing out of a transmitted mercurial taint progresses with greater rapidity than if occurring from the use of chloride of sodium. And now concerning the treatment.

First, allow me to remind you that from time to time you have heard what some have pleased to call my mere assertions to the effect that pyorrhoea alveolaris could not be cured by the removal of calcareous deposits or local treatment unaided.

Indeed you may have heard me deny the possibility of a cure, when the case was well developed and typically marked as such, but before proceeding further let me say right here that, from my standpoint, there is a vast difference between typical and non-typical cases. To me a typical case is one in which mercury in some shape or chloride of sodium has been a causation of conditions which have rendered possible the local manifestations regardless of tartar, fungus or other external irritants, conditions, which have their age and place of local evidence the same as any predisposition may grow into discrasia and bloom out into local symptoms and signs in accordance with its character and the parts so predisposed. Granting you that some of these cases in their earliest stages before much bony tissue has been involved and especially when the disease has been acquired or upon the other hand exists in the second or third generation from its origin can be checked or cured for a time.

We will now proceed with remedies.

Within the past decade almost every month, dental societies have mightily pondered over the different wonderful specifics for Riggs' disease, and our dental journals have regularly fed the hungry masses with alopathic doses of each and every new one. All kinds of powerful antiseptics have been on trial; germicides, parasiticides, escharotics, caustics, etc., are still in wholesale use. They may be of some service in their time and place, but after all is said and done there is but one principle upon which all local, *local*, I say, treatment should be based, *viz.*, that new tissue will not grow upon dead, in other words, broken nutrient continuity must have protection against substances inimical to the establishment of embryonic tissue.

Of these cases coming under the care of dentists the majority of them have passed the primary stage and the alveolar process has been more or less involved in the gradually advancing destruction; under these circumstances the first thing to be accomplished is the most thorough removal of all deposits of a foreign nature from about the necks, crowns, and roots of the teeth to the process. To do this completely and to assist you in the operation which follows, it often becomes necessary, in order that you may have room for the entrance and exit of instruments, to incise or split the gum, slightly, over or in the vicinity of the parts to be operated upon, especially is this required when the lesion exists between the teeth, and the septum is much broken down, for the chances are that the bone here will not be reproduced and by slitting the gums from external to internal aspect, you are not only enabled to work with more ease and certainty, but when the work is completed, the gum will more readily drop in over the parts separated, upon thus shielding against foreign substances, what reformation of tissue may take place.

During this operation it is proper from time to time to syringe well the parts with tepid salt-water, mixed with which may be any one of the good antiseptics now in common use. About as good a preparation as you will find for this purpose is listerine, which combines the qualities of an antiseptic with that of a stimulant and does not perceptibly coagulate albumen. When reasonably assured that the teeth are well cleaned your attention should next be directed to the removal of all disintegrating and lifeless bone. How easy to say and yet how seldom accomplished.

Indeed. this is often a very difficult and painful operation, not only so to perform, but doubly so to ascertain when it is complete. Months, even years, of experience, a steady hand, delicate sense of touch, a cool head, and a thorough knowledge of the parts are required. Aside from this you must be able to tell by the touch of an instrument the difference between the dead and living process.

There are but few instruments made that are well adapted for use in this operation. The ordinary scaler while suited to the removal of calcareous deposits from about the teeth is by no means of good form for the removal of dead bone. Six or seven different instruments will be sufficient. Of these two should have scoop points of different size; two, right and left curved chisel points, two sickle shaped blades and one a small slightly curved sounding instrument.

As in the preliminary operation, the use of the syringe may occasionally be continued after the removal of all lifeless bone and sometimes before it. Teeth much loosened from their sockets should be ligated with waxed silk thread to their fellow teeth. Following the operation an antiseptic in solution with teped water may be thoroughly used around about the teeth; but as for strong escharotics, caustics, and such like preparations, especially when used on pledgets of lint or cotton and to be left in the pockets for hours at a time, I think it entirely contrary to our best conceptions of the laws governing the reproduction of tissue. If the operation has been complete and the parts freed by syringing and the flow of blood from the debris resultant therefrom checked, then all has been done that can be of any avail, so far as you are concerned. Other duties devolve upon the patient.

On the other hand should the operation not be complete, all of the caustics, escharotics, antiseptics, germicides, and parasiticides known to ancient or modern times, in full strength, in solution, in crystals, in granules, amorphous or otherwise, on cotton, lint or silk, will be of as little use as an attempt to have the surgeon's flap unite over necrosed bone. What is needed after the operation is protection of the parts, and to my mind, nature's own, a clot of blood, is far superior to anything else.

The patient, as previously stated, should now have his duties to perform. He should be instructed to keep the teeth scrupulously free of food, to gently manipulate the parts by rubbing and pressing the gums with thumb and fingers, using the pressure inward toward the parts from whence bone has been removed, and after the soreness following the work has passed away, to use some kind of chewing gum, that the teeth and contiguous parts may be kept in gentle exercise.

In conclusion allow me to present a few questions still at issue, which deserve investigation if we desire a correct knowledge of the etiology of the ailment. First, do all persons who have deposits of salivary calculus about their teeth present the true characteristic signs and symptoms of this disease? If not, why? Second, is the disease infectious? If so, why have so very many of the human family escaped its ravages through centuries of contact and exposure to its action, and why are dentists who daily and hourly stand almost mouth to mouth with patients not also infected? Of course, we expect the reply from those who hold to the theory of infection that all persons are not alike susceptible to the action of forces and that as with all infectious disease one may be affected and others equally subject to the same producing causes show no sign of infection, the difference resulting from a different systemic condition. Very well. So far, so good.

Third, is the fact that the disease runs in families more an evidence of its infectious character than of its heredity?

Fourth, is it true that a peculiar and destructive fungus finds its habitat in the lesions of this malady and cannot be found in mouths not so affected?"

*Dr. Sage.* This is a subject upon which I profess to know little or nothing. I have had one case under treatment and the patient somehow or other got to using listerine as a mouth wash and the mouth improved under it.

*Dr. Berry.* It is very important for us when assuming to treat a case of pyorrheoa alveolaris, to look into the condition of the system; regulate it by a proper course of dietetics, see that the bowels discharge their functions and avoid an excessive use of common salt as it is undoubtedly a prominent cause of the disease. Scurvy among seafaring men is directly attributable to its inordinate use, as well as a lack of fruits and vegetables.

*Dr. J. Taft.* In our use of the agents mentioned by Dr. Harlan, there are certain principles by which we should be guided. There is the antiseptic principle, is it required? If so, then what is the proper one to be used in a given case?

If a mere disinfectant is required, we must determine what one is indicated according to its power. In cases where there is an acrid discharge taking place, a disinfectant merely, will suffice.

In considering these agents we must know all their properties; for instance, is an agent antiseptic, disinfectant, stimulant, or astringent? It may be any one of these or a number combined and this is the point we should determine when assuming to use it in the treatment of disease, if we propose to do so with intelligence. As to pyorrhoea alveolaris, there is a great difference of opinion upon the question of its being of systemic origin or a local affection merely.

When the constitution is good, the tissues good, nutrition well carried on and the elimination of waste matter properly performed, there will be but little constitutional or systemic predisposition towards the disease. If the disease *does* exist in this kind of a system, it is of purely local character.

If a system is made up of loose and flabby structures, with functions badly performed, it is then more susceptible to all kinds of affections, and, of course, to this one as well.

The nomination of the disease, *pyorrhoea alveolaris*, means pus oozing out from the margins of the gums; this can be produced by the presence of local irritants, which, if removed, will cause the disease to correct itself. Acrid secretions of the mouth, or acrid saliva, are sufficient to keep up the disease by getting into the pockets. The excretions from the mouth are sometimes very acrid, notably that of the saliva which is sometimes so vitiated that when coming in contact with the soft tissues it will burn them; consequently, it is an irritant fully capable of keeping up the disease by reason of this irritating property.

Common salt produces a change in tissues and bodies with which it comes in contact; it preserves meat, etc. In the system it prevents the free elimination of waste by preventing its free solution.

As Dr. Harlan is here now, I would like to ask him the *how* and the *why* concerning the use of some of the remedies and agents he mentioned this morning. What is the particular action of sanitas, is it purely antiseptic, astringent, stimulant or all and to what extent of each? I would also include eugenol.

*Dr. Harlan.* Sanitas stores up $H_2 O_2$; while not entirely recognized by dentists it is a germicide.

*Dr. Taft.* For all germs?

*Dr. Harlan.* For all bacterial forms and this is supported by the gentlemen I named in my paper.

Sanitas is a genuine disinfectant, it does not substitute one smell for another; when we speak of *disinfection* we do not mean the substitution of one smell for another. Its elements are capable of combining with the gas and other products of decomposition and so removing or destroying it. Sanitas is a powerful oxidizer. Sanitas is oxidized oil of turpentine; the turpentine oxidizes slowly, so that it takes a long time to produce sanitas. It does not give up its $H_2 O_2$ without a *call* for it, it does not do so spontaneously, so that in sanitas you have per-oxide of hydrogen and a dressing as well.

In teeth devitalized by a blow, my success has been greater with this agent than with anything else. A great many who complain of a want of success, do not get it on account of their carelessness. No outside matter must be permitted to enter the pulp chamber, all saliva, mucous, etc., should be thoroughly excluded. I never attempt to open a tooth unless it is dry. Some people do not like the taste of sanitas, in which case eugenol may be substituted. The reputation of the latter as a parasiticide rests upon solid facts. Put a mosquito germ into water and see how lively it is; drop upon him the minutest quantity of engenol, and he ceases to move; under its influence his whole alimentary canal is cleaned out.

Eugenol is a stimulant, a decided stimulant. It does not come under the head of disinfectants as sanitas does, it does not enter into combination with foul matter and break it up, its composition ($C_{10} H_{12} O_2$ if memory serves correctly) prevents that. Eugenol and water cannot be combined or used in connection. Sanitas remains where it has been injected, and does not mix with the saliva, though water may be used to slightly dilute it. Sanitas is made by passing a current of oxygen over oil of turpentine floating on water. I disguise sanitas by using it in conjunction with some of the aromatic essential oils.

The odor of iodoform may be disguised with eugenol very profitably, or, oil of peppermint may be used. Eugenol is more agreeable to the patient, but I prefer to open a pulp chamber and its canal or canals under sanitas; fill the cavity over the dressing of sanitas with gutta percha, puncturing it with two or three small holes to allow the escape of gas." (Adjourned.)

*Third Day—Last Session.*

The meeting was called to order at 10 o'clock a. m., with Second Vice President Emminger in the chair. The minutes of the second day's session were read by the Secretary, and on motion approved as read.

The third subject was passed, and the fourth, "Improvements in Prosthetic Dentistry," announced.

*Dr. Berry*—I saw a lady who had been a patient of Dr. Sheffield's, she had been under treatment for three months, and had the pleasure (?) of paying $500 for it. The piece of bridgework was perfect in every respect, the workmanship very beautiful. It was an upper denture, not quite full, and was clasped or fastened to two or three back teeth. I think the honesty of the thing was a damned humbug, as in a few years the dead roots to which it was fastened will give trouble.

*Dr. McKellops*—I have not done any artificial dentistry for fifteen years past, though I have seen a great deal of it. Dr. Patrick makes very beautiful bridgework which fits so tightly and perfectly, that when first put in, presses the blood out of the gum and turns it white. Some of the finest work I have ever seen came from his hands. I have also seen Sheffild's work in New York, one piece was a full upper denture attached to the two cuspids and two wisdom teeth; it set solidly in the mouth, and was efficient in mastication.

*Dr. H. A. Smith*—I have built up a tooth with oxyphosphate, using pins as anchorages. I also repaired a piece of Sheffield's work which had become loose on the molars to which it was attached. The objection to the method is the overwork of the teeth to which it is attached, causing periosteal inflammation from the irritation. In Dr. Patrick's method it seems to me that the accumulation of debris between the piece and the gums would result in irritation of the mucous surface.

*Dr. McKellops*—There is neither occasion nor opportunity for matter to get under the plate as it is perfectly fitted to the parts.

*Dr. H. A. Smith*—That is a remarkable statement, but I will not contradict it. It is very difficult to prevent matter from getting between the plate and the gum, no matter how tightly the plate fits. I would not do what Sheffield has done for $500, for $5,000, for I think it would have sent me to my grave. They must have had a time of it, but finally the dentist got the better of the patient and got the money too.

*Voice*—How much of a piece was the case to which you refer?

*Dr. H. A. Smith*—All the superior teeth were replaced except the two cuspids, two molars on one side and one on the other. The bands were somewhat loose, and the piece will have to be removed. This class of work cannot be done by the general practitioner on account of the time it consumes.

*Dr. Osmond*—All pulpless teeth to which crowns or bands are fitted, will eventually be forced from the socket by deposits within it, and the plate be lifted from the gums.

*Dr. McKellops*—I have seen some very beautiful work done by Dr. Morrison; one case was the capping of a molar, the cap being about half of the size of the tooth, it was so cemented on that there could be no friction; the work has worn for years. A man must be an artist to do such work as bridgework is capable of.

*Dr. F. W. Sage*—I don't know much about bridgework. The advocates of this kind of work do not use roots that have deposits on them, such as mentioned by Dr. Osmund, but select good teeth for anchoring and for the reason that so few opportunities are offered for using it, I do not think that the method will come into general use.

*Dr. Fletcher*—I would like to ask Dr. McKellops what is the condition of the mucous membrane after wearing Dr. Patrick's work?

*Dr. McKellops*—There is no inflammation at all, no disturbance.

*Dr. G. W. Smith*—I have had some experience with bridgework on the band method, and there seems to be no special inflammation caused by it.

*Dr. McKellops*—A case of Dr. Morrison's I wish to report. In an irregularity case, a front tooth struck the "stomach" tooth and caused pain and suffering. Dr. Morrison advised the extraction of both teeth, which was done. The "stomach" tooth was dressed and built up with gold to resemble the front tooth, an incisor, and placed into the socket which had been prepared for it with the burring engine. Some thought the operation could not be done, but it was, and successfully too, for the tooth has now been in about five months, and is as firm as any other in the mouth."

On motion the subject was passed; the Treasurer found the books at last, from which it appears that there was a balance of $33.84 in the treasury; the report was accepted and referred to an Auditing Committee. On motion of Dr. J. Taft the association went into the election of officers for the ensuing year with the following result:

President—A. F. Emminger, Columbus, O.

First Vice President—E. G. Betty, Cincinnati, O.

Second Vice President—Otto Arnold, Ironton, O.

Recording Secretary—A. G. Rose, Cincinnati, O.

Corresponding Secretary—W. D. Kempton, Cincinnati, O.

Treasurer—F. A. Hunter, Cincinnati, O.

On motion of Dr. Arnold the Secretary was voted $15, the janitor $5.

The Auditing Committee per Dr. Fletcher reported the accounts to be correct, and on motion of Dr. McKellops the report was accepted and adopted.

Dr. Betty presented bills amounting to $11.09 for printing and stationery, which were ordered paid, as also was Dr. Berry's account of $1 for postage.

Dr. F. A. Hunter was appointed by the chair as Master of Ceremonies to install the officers elect (neat and graceful speech from the new President, who handled the gavel with the familiarity of a veteran.) The retiring President Dr. Wm. Van Antwerp then delivered his address:

Gentlemen of the Mississippi Valley Dental Association, I will not try to edify you, as I feel incompetent, but as inexorable law has fixed the fact that your President must address you, I will try to make the infliction as anesthetic as possible. I propose to call your attention for a short time to some of the predisposing causes of dental disease.

Life is the sum of the functions that go to maintain it. When the system is complete in all its parts, and there is no interruption of the perfect performance of its functions we call this health, or sound physiological condition, but the human organism being very susceptible to various influences, as when there has not been perfect development of, or arrested function, and this carried to a point beyond endurance, then we have a diseased or pathalogical condition.

In the most healthful and correct performance of their functions, the several organs of the body necessarily suffer some waste of substance as well as expenditure of functional power, but while the general economy of nutrition is properly sustained the supply keeps pace with the demand. Excesses and irregularities, and every other means by which the constitutional laws and functional relations of the several organs are violated, not only is the whole system made to suffer, but the particular organs are made the seat of disease. By painful experience, most of the members of the human family, who have reached the age of twenty years, know that the teeth give evidence of the fact that some of Nature's laws have been violated. The tendency of heredity may be, and I think is, one of the greatest predisposing causes of disease. Unbalanced nutrition and secretion may be inherited. The most extensive and pernicious causes of disease of the teeth are those which manifest their effects through the general organic economy of the system. For instance, cows confined in stables, fed on still swill, hot and fermenting (a most unnatural diet), will in four years at most loose all their teeth. A physician who had traveled over nearly the whole globe informed me that he had observed the greatest difference in the teeth of different nations of men. Those that used much hot food and drink, and otherwise lived in unnatural conditions, possessed the worst, while on the islands of the Pacific those people who seldom or never take anything into the mouth hot, or used but little or no animal food, and otherwise lived in a simple natural way, have splendid teeth, and never suffer the pangs that Burns so graphically described. I have been among Indians, who, unalloyed by contact with the whites, knew no trouble with their teeth. The poor or working classes of Ireland and Scotland have excellent teeth. The negroes when newly arrived from Africa have good teeth, but after two or three generations residence among the whites they become even worse than those of their white neighbors.

I have also observed that those who live out in the open air a good deal have better teeth. As the log house is improved upon, the teeth deteriorate. The healthful effect upon the teeth of a full and regular performance of their natural function is much greater than generally supposed; for instance, any cause preventing mastication on but one side shows that the unused one suffers much more than the one in constant use. Imprison many of the wild animals and not allow them their natural food, their teeth will suffer, and even cause the death of the individual; in short, whatever produces a general disturbance of function, and causes a morbid irritability of the nervous system, attacks the teeth in common with all the other organs. We have seen that when proper mastication of food was accomplished, and thorough mixing of it with saliva, a part of the digestive function was consummated; now this shows how essential a good set of teeth is to the general economy. The American mode of eating as is generally seen in city hotels should be condemned, but it is thoroughly American to do the largest amount of everything in the shortest possible time. So to sum it up, it appears to me that as near as possible, we should run with a steady balance wheel, as no part of our organism can run eccentric without nearly all the apparatus suffering therefrom. High pressure and hot-house growing tells upon human systems, thereby not half living out their days as was intended. Nervous diseases new to the medical world are constantly being recognized. Only one year ago an M.D. friend of mine recognized an unnamed new nervous disease, and wrote to Dr. Hammond of New York, who classified it for him, and informed him that only thirteen cases had been so far recognized. Why is it there are so many tedious and dangerous cases of labor among our women, the poor classes furnishing few, notwithstanding they are obliged to work constantly up to the time of parturition? The Indians never to my knowledge. It is principally among the well-to-do classes, that do not have to work when disinclined. The false modesty that keeps a woman hidden the last three months of that eventful period causes more trouble than anything I know of, and this just when she needs God's pure sunlight and out-of-door exercise; just when to the organism of that new being, a healthy constitution may be imparted or not.

Anything that militates against a good sound constitution, to the same extent shows itself upon the teeth of the individual. To the dentist who from necessity or enterprise works eight or nine hours incessantly breathing the already depraved air, I say beware, you are laying the corner stone of your after misery in various ways. Take every opportunity to breath pure out-of-door air, exercise as your taste dictates. If you are beloved of honey bees plant an acre, your near neighbors will not rise up and call you blessed, but you will have profit in more than one sense; if you like gardening don't cultivate a beer garden, but one with at least one cabbage head in it; if you love manly sport, go lock up your business and your perplexities; go I say, yes, go and hunt and fish until you can sympathize with Esau, who coming in from hunting, tired and hungry, smelled the soup his brother was cooking, and sold his birthright for a plate full.

Your system will digest and assimilate more in one week while in some agreeable out door recreation, than it will during a month in troubles and perplexities. Those who do not know this by experience I pity, for it must be experienced to be enjoyed. Now if I have said anything or advanced a single idea that will help or encourage a brother, I will feel I have not spoken in vain, but so many ideas crowd upon each other in my mind that seem irrelevant, and may appear somewhat disjointed. I am much pleased with the progress of our profession, as there is a healthy activity shown in the whole field of dentistry, such a desire to find the foundation principles of every theory, that the man who speaks or writes on any subject is in jeopardy, if strong proof of his positson is not given. I thank you for your patient attention."

Dr. H. A. Smith offered this resolution which was unanimously carried:

"*Resolved*, Having learned that Professor Geo. Watt, one of the older and most efficient members of the M. V. D. A. being confined at home by sickness, the members desire to express regrets at his enforced absence, also to convey to him sympathy in his affliction, and express the hope that he may soon recover and be able to resume his accustomed professional work."

On motion of Dr. Moore a vote of thanks was tendered the retiring officers. Dr. Betty announced that there were several papers prepared for presentation before the association, but had not been reached, and desired to know what disposition be made of them; they were referred to the Committee on Publication for publication; the list includes papers by Dr. J. R. Callahan, G. D. Billings, F. W. Sage, and E. G. Betty. President Emminger then announced the following standing committees:

Executive—C. H. James, H. L. Moore, M. H. Fletcher.

Memcership—F. W. Sage, J. S. McCampbell, J. R. Callahan.

Publication—J. Taft, O. N. Heise, J. S. Cassidy.

Ethics—C. M. Wright, J. M. Clyde, G. W. Keely.

On motion of Dr. Clyde the President was instructed to appoint delegates to the American Dental Association. Adjourned to meet the first Wednesday in March, 1886.

